# External fixation in severe open tibial shaft fractures: indications, orthoplastic strategy, and infection-aware decision-making

**DOI:** 10.1530/EOR-2026-0107

**Published:** 2026-10-01

**Authors:** Sabri El Banna

**Affiliations:** ^1^ Centre Hospitalier Universitaire de Charleroi (HUMANI), Charleroi, Belgium; ^2^ Faculty of Medicine, Université libre de Bruxelles, Brussels, Belgium

**Keywords:** open tibial fracture, external fixation, intramedullary nailing, orthoplastic reconstruction, fracture-related infection, circular frame

## Abstract

- Intramedullary nailing remains the preferred definitive fixation for most open tibial shaft fractures once contamination is controlled and soft tissues permit internal implantation.
- External fixation should be chosen according to intent: damage control, preservation of orthoplastic access, staged bridge to internal fixation, or definitive frame-based reconstruction.
- Temporary frames are most useful in polytrauma, vascular injury, severe swelling, gross contamination, and wounds needing serial debridement, flap planning, or negative-pressure therapy.
- Conversion to intramedullary nailing is appropriate only when margins are declared, coverage is complete or reliably scheduled, contamination is controlled, and fracture-related infection (FRI) is not suspected.
- Definitive circular or hybrid fixation is favored when repeated excision, vascular repair, segmental bone loss, bone transport, uncertain margins, or suspected/established FRI makes buried metal less attractive.
- Successful frame treatment requires safe pin placement outside flap and implant corridors, restoration of length, axis, and rotation without distraction, timely soft-tissue coverage, and standardized infection and pin-site pathways.

## Introduction

Open tibial shaft fractures remain clinically demanding because skeletal stabilization, debridement, soft-tissue reconstruction, and infection prevention must be coordinated early. Locking intramedullary nailing has become the preferred definitive fixation for many open diaphyseal injuries when contamination is controlled and internal implants can be placed safely. However, severe soft-tissue compromise, vascular repair, segmental bone loss, repeated excision, or suspected fracture-related infection (FRI) still creates situations in which external fixation is strategically preferable rather than merely temporizing (1, 2, 3).

The difficulty is that ‘external fixation for open tibia’ is often discussed as if it were a single intervention, even though the same device family is used for four very different purposes: damage-control stabilization, staged orthoplastic reconstruction, planned bridge to internal fixation, and definitive frame-based reconstruction. This conceptual blurring partly explains why broad comparisons between external fixation and intramedullary nailing can be both statistically correct and clinically unhelpful.

The aim of this focused narrative review is not to relitigate whether external fixation or intramedullary nailing is universally ‘better’. Instead, it examines the modern role of external fixation in severe open tibial shaft fractures by answering three practical questions: when should a frame enter the treatment algorithm, when should it be converted to internal fixation, and when is definitive frame-based treatment the more coherent strategy? The clinical indications, technical principles, criteria for staged conversion, and practical decision algorithm are summarized in Tables 1, 2, 3 and 4, respectively.

**Table 1:** Clinical situations and the preferred role of external fixation.

Clinical situation	Preferred role of external fixation	Why this role fits
Polytrauma or physiologic instability	Temporary unilateral or biplanar frame	Provides rapid stabilization, safe transport, and protection of soft tissues while the patient is resuscitated
Severe open fracture awaiting serial excision or flap coverage	Temporary or staged frame	Maintains length and alignment while preserving wound access and reconstructive options
Vascular injury or vascular repair	Temporary frame; occasionally definitive frame if internal fixation threatens the repair	Stabilizes the limb quickly and protects the repair without adding buried metal across a hostile zone
Open fracture likely to convert later to intramedullary nailing	Temporary frame with planned conversion	Preserves future nail and incision corridors from the outset
Circumferential degloving, major contamination, or segmental bone loss	Definitive circular or hybrid frame	Avoids buried metal in a hostile bed and supports compression, shortening, relengthening, or transport
Established or suspected FRI	Definitive circular or hybrid frame when repeated access or bone reconstruction is required	Permits serial debridement, maintains stability, and supports infection-aware reconstruction

**Table 2:** Technical principles that determine success.

Technical principle	Why it matters	Common error to avoid
Place pins outside the zone of injury and outside likely flap or nail paths	Preserves reconstructive options and makes staged conversion feasible	Pins through future flap territory or implant corridor
Restore length, axis, and rotation without over-distraction	Protects soft tissues and creates biologically sensible reduction	A distracted frame or unrecognized malrotation
Match construct strength to treatment intent	Temporary and definitive frames have different mechanical goals	An underbuilt temporary frame that is simply left in place
Keep the frame compatible with wound care and vessel access	Allows repeat inspection, dressing changes, NPWT, vascular surveillance, and flap inset	A construct that blocks debridement or flap inset
Reassess perfusion, neurologic status, and compartments after application	Detects evolving ischemia, compartment syndrome, or reduction problems	Treating frame placement as the end of acute care
Standardize pin-site care and patient instructions	Improves surveillance and early treatment of frame-related problems	Inconsistent follow-up or vague home-care advice

**Table 3:** Features favoring staged conversion to intramedullary nailing versus definitive frame-based treatment.

Decision feature	Favors staged conversion to IM nailing	Favors definitive external fixation
Soft-tissue viability	Viability clear; definitive cover completed or reliably planned	Ongoing necrosis, repeated excisions, or uncertain soft-tissue margins
Contamination or infection concern	Contamination controlled; no clinical concern for infection	Persistent contamination or established/suspected FRI
Bone defect	No major bone loss requiring transport or staged correction	Segmental bone loss or need for compression, shortening, relengthening, or transport
Reconstructive access	Internal fixation corridor available without compromising cover	Flap design, vascular repair, or wound access would be compromised by internal implants
Strategic goal	Load-sharing fixation and simpler rehabilitation after soft-tissue recovery	A frame-based reconstructive strategy remains the safest and most coherent pathway

**Table 4:** Practical decision algorithm for contemporary external fixation use.

Step	Key question	If yes	If no
1	Does the patient or limb require rapid provisional stabilization?	Apply a temporary external fixator after debridement and document the planned next step	Proceed to definitive fixation assessment after debridement
2	Does the wound require serial look operations, flap planning, NPWT as a bridge, or unrestricted soft-tissue access?	Continue staged external fixation within an orthoplastic pathway	Assess whether definitive internal fixation is already safe
3	Are contamination controlled, margins declared, coverage complete or reliably planned, and FRI not suspected?	Convert in a planned manner to intramedullary nailing or another internal construct	Continue frame-based treatment until biology and soft tissues are ready
4	Does the limb require infection-focused reconstruction, vascular protection, compression, bone transport, or prolonged access to the fracture zone?	Use definitive circular or hybrid external fixation	Choose internal fixation when it offers simpler and safer definitive care

## Narrative review approach

This article was designed as a focused narrative review for clinical decision-making rather than as a systematic review or pooled meta-analysis. PubMed/MEDLINE was searched from database inception to April 17, 2026, using combinations of the terms ‘open tibia’, ‘open tibial fracture’, ‘tibial shaft fracture’, ‘external fixation’, ‘ring fixator’, ‘circular frame’, ‘hybrid fixator’, ‘intramedullary nail’, ‘damage control’, ‘orthoplastic’, ‘flap coverage’, ‘negative-pressure wound therapy’, ‘vascular injury’, ‘bone loss’, ‘bone transport’, ‘pin-site infection’, and ‘fracture-related infection’. Reference lists of included articles were hand-searched, and official guideline websites were reviewed for contemporary open-fracture standards.

Sources were eligible when they addressed adult open diaphyseal tibial fractures or a directly transferable principle of open-fracture care, including fixation strategy, staged conversion, orthoplastic access and coverage, contamination, vascular repair, bone loss, external ring or hybrid fixation, FRI diagnosis or treatment, and pin-site surveillance. Pediatric fractures, periarticular fractures, tibial pilon or plateau injuries, elective deformity correction, and non-tibial fracture studies were excluded unless they clarified an acute trauma principle directly applicable to the tibial shaft.

The final evidence base was selected by clinical relevance to the decision points of this review. Priority was given to open-fracture guidelines, international FRI consensus documents, randomized trials, meta-analyses, and cohort studies of adult open tibial shaft fractures. Seminal older papers were retained when they continued to inform orthoplastic timing or frame mechanics. Because the aim was interpretive synthesis, no formal PRISMA workflow, database-level duplicate counting, or quantitative risk-of-bias scoring was undertaken; this limitation is acknowledged when interpreting comparative evidence.

## Where external fixation enters the algorithm

The first decision is not whether the surgeon ‘likes frames’ or ‘likes nails’; it is what the patient and limb require at that stage. External fixation should be considered when one or more of the following are present: physiologic instability or polytrauma requiring rapid provisional stabilization; soft-tissue injury that needs serial assessment, serial debridement, or flap planning; vascular injury or repair that should be protected without adding buried metal; contamination or doubtful margins that make immediate internal implantation unattractive; bone loss or shortening that points toward a frame-based reconstructive strategy; and established or strongly suspected FRI (1, 2, 4, 5, 6).

Framed this way, external fixation becomes an intent-specific tool rather than a default ideology. A unilateral half-pin frame used during the first 24–72 h after debridement is not the same treatment as a definitive circular construct used for weeks or months. The surgeon must therefore decide early whether the frame is meant to rescue physiology, preserve reconstructive access, prepare for staged conversion, or serve as the definitive platform for compression, transport, or infection-focused reconstruction. Once the intended role is explicit, pin placement, construct geometry, and rehabilitation become more logical.

## Damage-control and early provisional stabilization

In polytrauma, major swelling, vascular injury, or heavily contaminated wounds, temporary external fixation remains one of the fastest ways to restore gross length and alignment, facilitate transport, and protect the soft tissues after debridement. Its value in this setting is less controversial than its value as definitive fixation. The immediate goals are reduction to a biologically sensible length, prevention of additional soft-tissue trauma, access for repeat excision, and preservation of options for vascular and plastic reconstruction (1, 2).

The temporary frame must be planned with the next operation in mind. Pins inserted through future flap territory, future incisions, or the anticipated nail corridor can convert a good salvage strategy into an avoidable reconstructive problem. Surgeons who expect later intramedullary nailing should place pins as though conversion were already scheduled. Over-distraction is a recurrent error. A frame that restores gross length but leaves the fracture distracted can increase soft-tissue tension, impede contact biology, and compromise vascular or flap work. Reduction after frame application should therefore be documented in terms of length, axis, and rotation, rather than assumed adequate because the limb looks straight from a distance (7, 8, 9, 10).

## External fixation within an orthoplastic pathway

For severe open tibial fractures, fixation strategy and soft-tissue strategy cannot be separated. Contemporary standards emphasize intravenous antibiotics as early as possible, combined orthopedic–plastic planning, formal wound excision, and definitive soft-tissue cover at a specialist orthoplastic center when required (1, 2, 3). External fixation often fits this pathway well because it maintains alignment while keeping the wound accessible for serial inspection, vessel work, negative-pressure wound therapy (NPWT), skin grafting, or flap inset. NPWT should be regarded as a temporary adjunct between debridement and closure or coverage, not as a reason to delay definitive soft-tissue reconstruction when the limb is ready.

Older microsurgical literature established the importance of early coverage, and modern protocols continue to support rapid coordinated care (11, 12). The classic ‘fix and flap’ concept remains relevant, but the key contemporary message is organizational: the limb does best when debridement, fixation, coverage, and infection prevention are planned as a single pathway rather than as isolated procedures. This is also where a properly placed frame can be more useful than immediate internal fixation – not because internal fixation is inferior in principle, but because the frame can preserve access and avoid interfering with flap design or repeated wound management.

Timing still matters. BOAST and BOA/BAPRAS standards recommend early antibiotics and coordinated orthoplastic management, with definitive soft-tissue closure or coverage as soon as feasible, ideally within 72 h (1, 2). More recent cohort data suggest that when immediate coverage is not achievable, delay still matters and outcomes worsen as soft-tissue reconstruction is pushed later (13, 14). The practical lesson is not dogmatic adherence to a single clock, but disciplined avoidance of unnecessary delay.

## Planned conversion to internal fixation

Many frames applied to open tibial fractures should be understood from the outset as stage 1 of a staged strategy rather than as a reluctant compromise. Planned conversion to intramedullary nailing is attractive when contamination has been controlled, wound margins are declared, soft-tissue coverage is complete or reliably scheduled, and no clinical features suggest FRI. In that setting, internal fixation can reduce the risk of late malalignment, simplify rehabilitation, and allow the definitive implant to function as a load-sharing device rather than as a buried foreign body in a hostile bed (15, 16, 17, 18, 19, 20, 21, 22).

The key word is planned. Pin placement must preserve the intended nail entry point and trajectory, and the temporary construct should provide enough stability to avoid secondary displacement before conversion. There is no universally valid ‘safe day’ on which all frames should be converted. Instead, timing should be guided by wound biology, certainty of coverage, and infection risk. Nonetheless, recent data suggest that longer duration of provisional external fixation and higher Gustilo severity are associated with higher infection risk after conversion, reinforcing the importance of efficient progression once the limb is ready (7).

Planned conversion should not be portrayed as treatment failure. In severe open fractures, staged fixation can be the most coherent way to balance biology and mechanics.

## When definitive frame-based reconstruction is the better option

The indication for definitive frame-based reconstruction becomes strongest when the limb remains biologically, mechanically, or infectiously hostile after the initial phase. This includes circumferential degloving, major periosteal stripping, severe contamination, segmental bone loss, vascular repair, repeated excisions, uncertain soft-tissue margins, or established/suspected FRI. In these situations, avoiding buried implants can simplify repeated debridement, preserve wound and flap access, and support compression, shortening, relengthening, deformity correction, or bone transport (4, 5, 6, 22).

Circular or hybrid fixation offers specific advantages when the reconstructive plan requires adjustable multiplanar alignment, gradual compression or distraction, staged docking-site management, bone transport, or ongoing access to the fracture zone. These advantages are not provided by a simple unilateral temporary frame and are not always provided by intramedullary nailing. The clinically relevant question is therefore not ‘frame or nail?’ in the abstract, but whether the construct actively serves the limb’s soft-tissue, vascular, bone-defect, and infection strategy (9, 10, 21, 22).

Definitive external fixation is therefore most defensible when the frame is chosen because it actively advances the treatment goal, not because internal fixation has been rejected reflexively. A frame that preserves flap freedom, protects a vascular repair, or enables compression and transport is serving a purpose that an intramedullary nail may not.

A practical threshold is useful: definitive external fixation is most defensible when at least one of three conditions is present. First, internal fixation would compromise soft-tissue reconstruction or vascular repair. Second, bone loss or shortening requires compression, relengthening, or transport. Third, suspected or established FRI requires serial debridement, deep sampling, dead-space control, and reconstruction without adding or retaining internal metal when that is judged undesirable.

## Infection-aware decision-making and FRI

Infection-aware decision-making means that fixation is chosen after explicitly considering infection probability, the need for repeat debridement and deep tissue sampling, dead-space management, timing of soft-tissue coverage, and systemic or local antimicrobial strategy. It is not simply the selection of the implant with the lowest average infection rate in routine comparative trials. The international FRI consensus provides standardized definitions and a diagnostic framework based on confirmatory and suggestive criteria (4, 5). Once FRI is suspected or established, fixation choice must be integrated with debridement strategy, deep tissue sampling, dead-space management, soft-tissue coverage, and antimicrobial treatment (6).

In this context, external fixation has a clear contemporary role. It allows repeated access for debridement, avoids adding or retaining internal metal in a compromised bed when that is judged undesirable, and supports compression or transport when debridement leaves bone loss. That does not mean external fixation is always superior for infection prevention in acute open fractures; it means it remains strategically useful when infection dominates the reconstructive problem. Fracture stability still matters in FRI, but so does the ability to re-enter the wound safely and repeatedly.

## What comparative evidence actually shows

The comparative literature should be interpreted with greater nuance. Older meta-analyses and newer systematic reviews broadly agree that intramedullary nailing performs at least as well as, and often better than, external fixation for many open tibial shaft fractures, particularly with respect to superficial infection and malalignment (15, 16, 17, 18, 19, 20, 21). A network meta-analysis reported lower unplanned reoperation with intramedullary nailing compared with external fixation (18), and two 2023 meta-analyses confirmed lower rates of superficial infection and malunion with nails (19, 20). A 2024 meta-analysis further concluded that intramedullary nailing is generally preferred for Gustilo–Anderson I–IIIA fractures, while noting that ring fixators compare more favorably than ordinary external fixators within this subgroup (21).

Randomized trials tell a similarly qualified story. In Tanzania, intramedullary nailing and external fixation produced similar rates of the primary composite outcome and of deep infection at one year, but intramedullary nailing achieved better radiographic alignment and earlier healing signals (17). The FIXIT trial, which compared modern ring fixation with internal fixation for severe open tibial fractures, argued against routine default use of ring fixation by showing more major limb complications, largely driven by loss of reduction and implant-related problems, while deep infection rates were not clearly different (22).

The key limitation is external validity for the most severe reconstructive subgroups. Comparative trials are most informative for routine, lower-grade, or standardizable injuries. They are less able to answer the decisions faced in Gustilo–Anderson IIIB or IIIC injuries requiring flap-based reconstruction, vascular repair, serial excision, segmental bone-loss management, bone transport, or infection-focused surgery. Such limbs are often excluded, uncommon, heterogeneously treated, or analyzed without enough detail to separate temporary external fixation, routine definitive external fixation, and planned circular-frame reconstruction.

This distinction changes the conclusion. Existing comparative evidence argues against using external fixation as the routine definitive treatment for all open tibial shaft fractures. It does not eliminate the value of selected external fixation when the treatment goal is orthoplastic access, vascular protection, staged conversion, bone-defect reconstruction, or FRI management. A defensible review must therefore compare strategies and indications, not devices in isolation.

## Technical principles that determine success

The performance of external fixation in open tibial trauma depends less on rhetoric than on execution. Several technical principles recur across modern practice and should be made explicit.

First, pin and wire placement must respect both biology and future surgery. Pins should be placed outside the zone of injury, away from likely flap territory, outside planned incision lines, and outside any anticipated path for later intramedullary or plate fixation.

Second, construct geometry should match intent. Temporary frames need rapid, reliable provisional stability, whereas definitive frames may require multiplanar control, dynamization, or the ability to compress or transport. Frame strength is influenced by pin spread, rod-to-bone distance, the number of fixation levels, and ring geometry (8, 9, 10).

Third, reduction goals must be explicit. Restoring length, axis, and rotation without harmful distraction is more important than applying a technically neat but biologically unsound construct. Small residual cortical gaps in massive soft-tissue injury may be acceptable; persistent distraction is not.

Fourth, the frame should preserve function whenever possible. Prolonged joint spanning, obstructed wound access, and frames that prevent flap inset or vessel surveillance should be avoided unless they are unavoidable in the acute phase.

Finally, pin-site surveillance should be standardized. Pin-site irritation and infection remain the most common frame-related complications. Contemporary definitions help distinguish benign irritation from true infection, and prevention still rests on good insertion technique, stable fixation, consistent aftercare, and early response to drainage, pain, or skin change (23, 24).

## Discussion

The contemporary role of external fixation in severe open tibial shaft fractures is narrower than it was in the pre-locking-nail era, but it remains clear and important. The mistake is to discuss external fixation as either obsolete or universally indispensable. It is neither. Intramedullary nailing is the preferred definitive fixation for many open diaphyseal tibial injuries once contamination and soft-tissue conditions permit internal implants. External fixation becomes most valuable when the limb’s immediate needs are provisional stabilization, preserved access for orthoplastic care, staged conversion, or definitive reconstruction without buried metal.

This focused formulation avoids two errors: i) overclaiming that external fixation is essential for open tibial fractures in general and ii) dismissing it as obsolete because many routine fractures do well with intramedullary nailing. The relevant modern role is narrower but clinically important: external fixation is most valuable when the limb needs rapid provisional stability, preserved access for orthoplastic care, planned conversion after biological readiness, or definitive reconstruction without buried metal.

The limitations of the literature remain important. Many randomized trials are older, use frame constructs that do not represent current circular fixation practice, or enroll injuries that are less reconstructively complex than the cases in which contemporary surgeons most value frames. Conversely, single-center orthoplastic series and FRI cohorts may better represent severe subgroups but are less amenable to pooled comparison. The practical implication is that treatment should be driven by injury pattern and reconstructive intent rather than by simplistic device competition.

Future studies should compare strategies that surgeons actually face: staged external fixation followed by nailing after flap coverage versus primary nailing in flap-worthy injuries; definitive ring fixation versus internal fixation in bone loss and vascular repair; and implant-retaining versus frame-based approaches within standardized FRI pathways.

## Conclusion

External fixation should not be framed as the routine definitive treatment for all open tibial shaft fractures, nor dismissed as a historical alternative to intramedullary nailing. In severe open diaphyseal injuries, its contemporary value lies in damage-control stabilization, orthoplastic pathway management, planned staged conversion, and definitive reconstruction when contamination, vascular repair, bone loss, or FRI makes internal implants less attractive. The best results come when the role of the frame is defined from the outset and integrated with soft-tissue cover, infection strategy, and future reconstruction.

## ICMJE Statement of Interest

The author declares that there are no conflicts of interest related to this review.

## Funding Statement

This work did not receive any specific grant from any funding agency in the public, commercial, or not-for-profit sector.

## Author contribution statement

The article was completely written by the author.

## Data availability

No new data were generated or analyzed for this narrative review.

## Ethics approval and informed consent

Not applicable for a narrative review.
